# Case Report: Trendelenburg gait caused by retained drain fragment: a rare complication of total hip arthroplasty

**DOI:** 10.3389/fsurg.2024.1519414

**Published:** 2025-01-06

**Authors:** Selahaddin Aydemir, Ozgur Aydin, Mustafa Çeltik, Burak Duymaz, Mehmet Erduran

**Affiliations:** ^1^ Orthopedics and Traumatology Department, Kastamonu Training and Research Hospital, Kastamonu, Türkiye; ^2^ Orthopedics and Traumatology Department, Dokuz Eylül University, Izmir, Türkiye; ^3^ Orthopedics and Traumatology Department, Dr. Abdurrahman Yurtaslan Ankara Oncology Training and Research Hospital, Ankara, Türkiye

**Keywords:** drain management, orthopedic surgery complication, Trendelenburg gait, gluteal tendinopathy, retained drain

## Abstract

Retained drain fragments, though rare, can lead to significant complications in orthopedic surgery(1). This case report presents a 57-year-old woman who developed gluteal tendinopathy and Trendelenburg gait two years after a total hip arthroplasty (THA) due to a retained drain fragment. A less experienced surgeon encountered resistance during drain removal on the first postoperative day and applied excessive force, unknowingly leaving a fragment inside. The patient initially had no symptoms, but later presented with pain and gait disturbances. Radiographic evaluation revealed the retained drain, necessitating surgical removal and gluteus medius augmentation. The patient subsequently underwent a structured rehabilitation program. This case emphasizes the importance of careful drain management, proper postoperative evaluation, and collaborative patient-doctor decision-making to prevent such complications.

## Introduction

The use of drainage systems in orthopedic surgery remains a controversial yet common practice, primarily aimed at removing blood and seroma from the surgical field during the postoperative period (1). Although generally safe, drain removal occasionally poses challenges, particularly when resistance is encountered, as inexperienced surgeons may inadvertently leave a fragment behind (2).

While cases of retained drains have been reported in various surgical fields (3–6), their long-term complications in orthopedic surgery are less frequently documented. Potential outcomes range from asymptomatic retention to severe issues like limited range of motion (ROM) and cartilage damage (7–11). This case report highlights an unusual complication: gluteal tendinopathy resulting in Trendelenburg gait due to a retained surgical drain fragment, a scenario not previously reported in the literature. This report underscores the critical need for meticulous drain management and postoperative evaluations to prevent similar outcomes.

## Case report

A 57-year-old female patient, with a body weight of 70 kg and a height of 165 cm, underwent a total hip arthroplasty due to a left femoral neck fracture. This surgical intervention involved the precise placement of a prosthetic implant to restore the functionality of the hip joint, as well as a meticulous repair of the abductor mechanism to ensure proper hip stability and movement post-surgery. To effectively manage the risk of postoperative hematoma formation, a 16F Hemovac drain was strategically inserted beneath the fascia. This drain serves to evacuate any excess blood or fluid that may accumulate in the surgical area, thereby promoting a smoother recovery process and reducing the likelihood of complications. The closure of the surgical site was performed by a less experienced surgeon, albeit under the close supervision of a more seasoned surgical professional. This collaborative approach not only aimed to enhance the surgical outcome but also provided an invaluable learning opportunity for the less experienced surgeon, ensuring that the procedure was conducted with the highest standards of care and precision.

### Postoperative course

**Figure 1:**
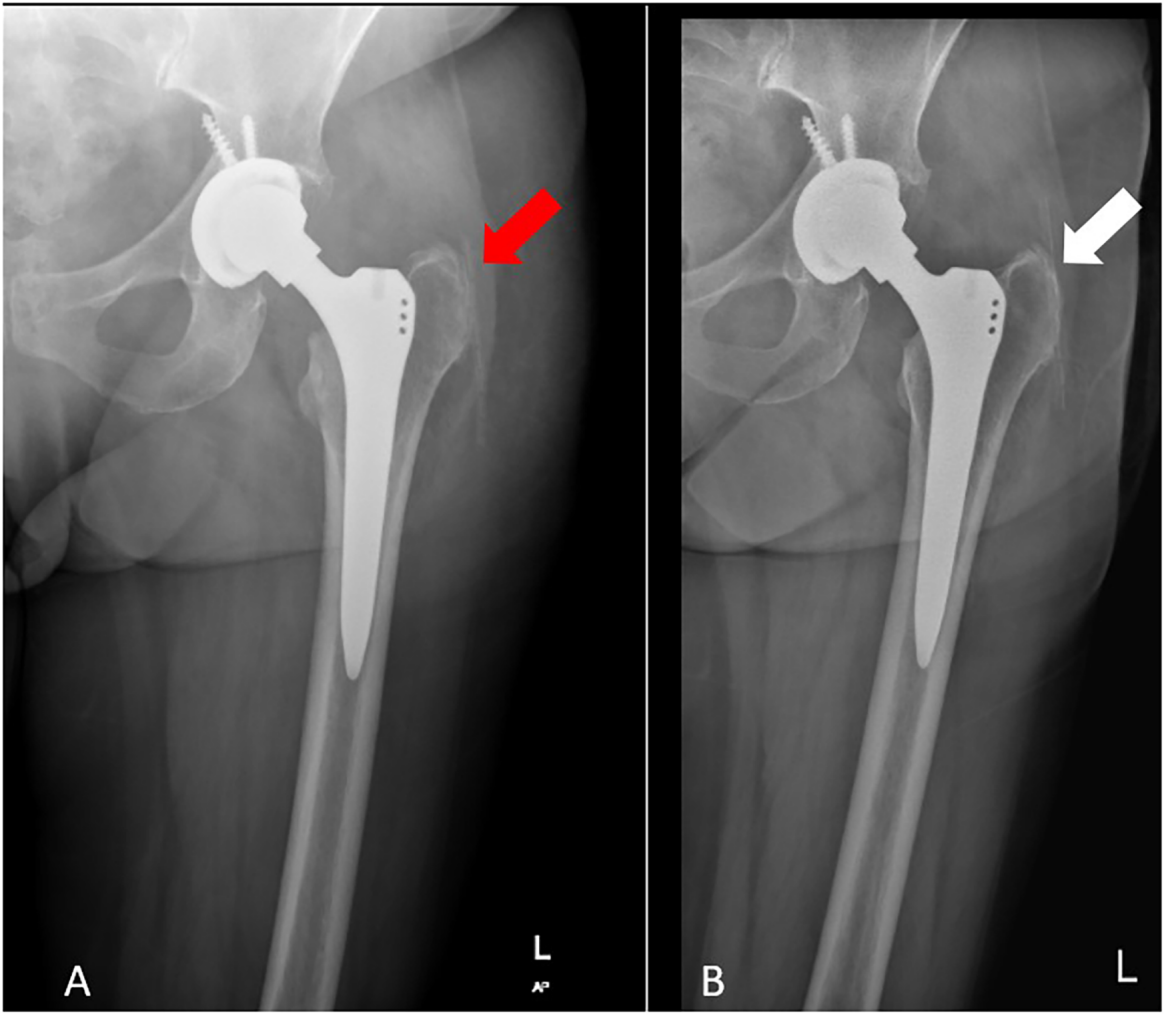
**(A)** Initial postoperative radiograph showing misdiagnosed retained drain (limited view). **(B)** X-ray showing the retained drain fragment two years post-surgery.

On the first postoperative day, a significant challenge arose during the removal of the surgical drain. The less experienced surgeon, encountering unexpected resistance, mistakenly assessed the situation as a case of the drain being stuck. In an attempt to resolve this issue, the surgeon applied excessive force to extract the drain, inadvertently leaving a fragment of it embedded within the patient's body. At postoperative day, standard radiographic assessments(pelvis ap, femur ap and femur lateral x-ray) were performed to monitor the alignment and possible periprosthetic fracture. However, it is crucial to note that an anterior-posterior (AP) pelvic x-ray, which is instrumental in identifying potential soft tissue abnormalities, was not conducted. Specifically, it refers to an anterior-posterior (AP) pelvic x-ray, which is routinely performed to evaluate both the alignment and placement of implants and to identify any soft tissue abnormalities. In this case, however, the initial radiographic assessment was incomplete as it did not include sufficient views to evaluate the entire surgical area comprehensively, particularly the soft tissue. This oversight proved significant, as the retained fragment was overlooked during this clinical early stage of recovery (Figure 1A). Despite this unrecognized complication, the patient was mobilized later that same day, and her initial recovery trajectory appeared to be progressing without any notable issues. As the second week of recovery commenced, the patient returned to the clinic for the routine removal of sutures. The combination of inadequate imaging protocols and the high volume of patients in the outpatient clinic contributed to this unfortunate lapse, allowing the complication to remain undetected and potentially complicating the patient's recovery journey.

### Symptom progression

Two years following the surgical intervention, the patient returned for evaluation, reporting ongoing and debilitating pain localized to the left hip, accompanied by a noticeable instability in their gait, characteristic of a Trendelenburg gait. This particular gait pattern, marked by a drop of the pelvis on the opposite side during ambulation, suggested underlying issues with hip stability and muscle function. Upon conducting a thorough clinical assessment and engaging in detailed dialogue with the patient, it became evident that the discomfort had intensified over time, with the patient also expressing a peculiar sensation akin to the presence of a foreign object within the hip area. To investigate these symptoms further, a series of repeat radiographic imaging studies were performed, which ultimately revealed the unexpected presence of a retained fragment of a surgical drain, as illustrated in Figure 1B. This finding raises significant concerns regarding postoperative complications and the need for potential intervention to address the retained material and alleviate the patient's ongoing symptoms.

### Surgical intervention

**Figure 2:**
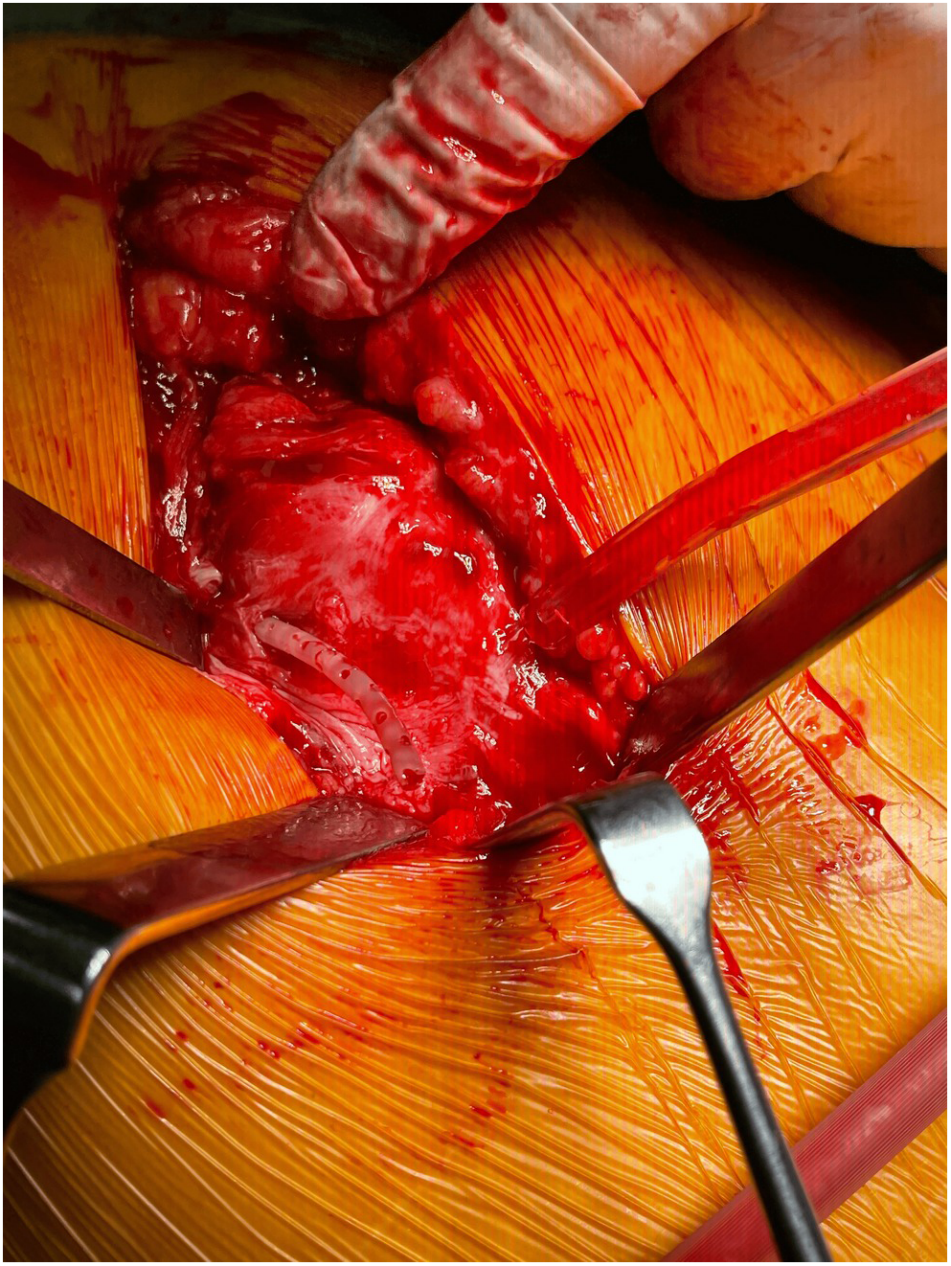
Surgical findings of tendinopathy and retained drain fragment entangled in muscle fibers. Appearance compatible with gluteal tendinopathy due to chronic irritation.

The surgical intervention was meticulously planned to address the retained fragment, following a preliminary diagnosis of gluteal tendinopathy. The procedure was conducted under general anesthesia to ensure the patient's comfort and safety. The surgical team carefully reopened the previous incision, allowing for direct access to the affected area. Upon exploration, it was discovered that the retained fragment approximately 5 cm long was intricately entangled within the fibers of the gluteus muscle, presenting a challenge that required precise dissection and careful handling to avoid further damage to the surrounding tissues (Figure 2). The fragment was successfully excised, alleviating the potential for ongoing irritation and dysfunction. In addition to the removal of the fragment, the surgical team addressed the underlying tendinopathy at the tendon insertion site, which is critical for restoring optimal function. This was achieved through anchor augmentation, a technique that enhances the stability of the tendon attachment and promotes healing. The augmentation process involved the placement of anchors that secure the tendon more effectively to the bone, facilitating a stronger and more durable repair.

Following the surgical procedure, the patient was placed on a comprehensive six-week structured rehabilitation program. This program is designed to promote recovery, restore strength, and improve flexibility in the affected area, allowing for a gradual return to normal activities while minimizing the risk of re-injury. The rehabilitation regimen includes targeted exercises, physical therapy, and regular assessments to monitor progress and adjust the program as necessary for optimal recovery outcomes. At the six-month follow-up, she reported significant improvement in mobility and resolution of Trendelenburg gait. However, mild residual tendinopathy symptoms persisted, managed with ongoing physiotherapy.

## Discussion

Retained drain is a rare and preventable complication that can be stressful for the patient and the surgeon (9). Most surgeons prefer to explore and remove the retained drain due to concerns about malpractice, legal issues, and potential complications. However, this will require an additional risk of anesthesia and a new surgical procedure. Therefore, should the part of the drain retained in the patient be removed? The question remains the subject of debate.

Different drain fixation methods have been suggested to minimize these preventable complications (9, 12). For a younger surgeon on the learning curve, the risk of complications is higher, along with many environmental factors such as carelessness and fatigue. To manage in review this complication effectively, it is crucial to ensure proper fixation of the drain after closing the layers, observe its movement, and detect any issues early.

Although undesirable, cases and complications related to retained drains are reported across various surgical fields (3–6). In orthopedic surgery, cases where no complications were reported in the long-term results for drains broken in the soft tissue and sometimes in the joint have been shown (7, 8, 10). In the report of 2 cases published by Cox et al., the drain retained in the knee joint was removed due to severe pain and limitation of movement (9). In contrast, the retained drain in the retzius space provided an uncomplicated recovery (9). In orthopedics, foreign bodies in joints can cause cartilage damage, restricted range of motion, and pain, making their removal advisable (1, 13). Gupta et al. reported a case of ROM limitation and cartilage damage, which affected the quality of life in the postoperative fifth month due to retained drain after knee arthroscopy (11). There are no controlled studies on managing retained drains in orthopedics, and only a limited number of case examples exist. At this stage, the doctor's cooperation with the patient is essential in treatment management. Limited examples in the literature indicate that drains retained in soft tissue are generally followed, and drains retained in the joint may cause mechanical problems and may need to be removed. However, this case highlights that extra-articular drains are not without issues either.

Gluteal tendinopathy is the most common cause of primary lateral hip pain, with many identified risk factors (14). It causes many clinical symptoms, including pain, tenderness, and gait disturbance in advanced stages (14, 15). Trendelenburg gait is characterized by a compensatory twist or wobbling towards the pathological side to balance the body's center of gravity, which occurs due to the disorder of the hip abductor mechanism. One acquired cause of this gait is gluteal tendinopathy (16, 17).

In our case, chronic irritation and delayed wound healing were caused by the internal drain, which led the patient to return with pain and gait disturbances. Since we do not have a similar example, we report this case of gluteal tendinopathy causing Trendelenburg gait as a complication of the retained drain.

### Discussion key findings

This case serves as a crucial reminder of the potential complications associated with retained drains, a scenario that, while infrequent, can result in severe and lasting consequences such as chronic irritation of surrounding tissues, the development of tendinopathy, and disturbances in normal gait patterns. The case emphasizes that timely detection and appropriate management of these complications could have significantly reduced their severity. This underscores the vital importance of implementing postoperative imaging protocols and conducting comprehensive clinical evaluations to identify issues early on, ultimately promoting better patient outcomes.

### Role of experience and supervision

The complications observed in this case were exacerbated by the inexperience of the less experienced surgeon involved. This situation highlights the essential role that senior surgeons play in the training and supervision of less experienced medical staff. Their guidance is crucial in minimizing the risks associated with surgical procedures. Furthermore, it is imperative that proper techniques, such as the careful application of force during the removal of drains and thorough intraoperative checks, are consistently practiced to ensure patient safety and minimize the likelihood of complications.

### Lessons learned

#### Drain management

The implementation of standardized protocols for drain management is essential. This includes practices such as the trimming of drain tips to prevent obstruction and ensuring that all drains are completely removed before concluding a surgical procedure. Such measures can significantly reduce the risk of drain retention and its associated complications.

#### Postoperative imaging

The routine use of anteroposterior (AP) radiographs in the postoperative period is recommended to facilitate early detection of any abnormalities. This proactive approach can help identify issues before they escalate into more serious complications, allowing for timely intervention.

#### Patient-doctor collaboration

Engaging in shared decision-making with patients is vital for effective management of complications. By fostering open communication and ensuring that patients are well-informed about their options, healthcare providers can enhance the overall quality of care and improve patient satisfaction. This collaborative approach not only empowers patients but also promotes adherence to treatment plans, ultimately leading to better health outcomes.

## Patient perspective

The patient expressed significant relief following the removal of the retained drain fragment, reporting improved mobility and a reduction in pain. Initially, the patient was frustrated by the delayed diagnosis, attributing her prolonged discomfort and gait disturbance to unaddressed surgical complications. However, she appreciated the clear communication and collaborative approach during the second surgical intervention and rehabilitation process. The patient emphasized the importance of comprehensive follow-up care and detailed postoperative imaging, noting that earlier identification of the retained drain fragment could have alleviated her symptoms sooner. Overall, she is satisfied with her current condition and remains optimistic about the continued benefits of physiotherapy in managing residual tendinopathy.

## Conclusion

This case highlights the need for vigilance in drain management and postoperative evaluations. Retained drains, though preventable, can lead to severe complications such as gluteal tendinopathy and Trendelenburg gait. Adhering to standardized surgical protocols, providing adequate training to less experienced surgeons, and fostering collaborative decision-making are essential steps in improving patient outcomes.

## Data Availability

The datasets presented in this article are not readily available because this is a case report. Requests to access the datasets should be directed to Selahaddin Aydemir, selahaddinaydemir@gmail.com.
